# Association between Harmful and Addictive Perceptions of E-Cigarettes and E-Cigarette Use among Adolescents and Youth—A Systematic Review and Meta-Analysis

**DOI:** 10.3390/children9111678

**Published:** 2022-10-31

**Authors:** Ameera Syafiqah Aly, Rokiah Mamikutty, Jamaludin Marhazlinda

**Affiliations:** 1 Department of Community Oral Health and Clinical Prevention, Faculty of Dentistry, Universiti Malaya, Kuala Lumpur 50603, Malaysia; 2 Oral Health Program, Ministry of Health, Putrajaya 62590, Malaysia

**Keywords:** e-cigarette, risk perception, harmful, addictive, adolescent, youth, young people

## Abstract

Background: Recognising the association between the perceived risks of e-cigarettes and e-cigarette usage among youth is critical for planning effective prevention and intervention initiatives; thus, a systematic review and meta-analysis were performed. Methods: Fourteen databases were searched for eligible studies from the Inception of database until March 2022 to examine the effect estimates of the association between perceptions of harmfulness and addictiveness and overall e-cigarette usage among adolescents and youth. Results: The meta-analysis showed that in comparison to non-users, young people who were ever e-cigarette users were two times more likely to disagree that e-cigarettes are harmful (OR: 2.20, 95% CI: 1.41–3.43) and perceived e-cigarettes as less harmful than tobacco cigarettes (OR: 2.01, 95% CI 1.47–2.75). Youths who were ever e-cigarette users were also 2.3 and 1.8 times more likely to perceive e-cigarettes as less addictive (OR: 2.28, 95% CI: 1.81–2.88) or perceive e-cigarettes as more addictive (OR: 1.82, 95% CI: 1.22–2.73) than tobacco cigarettes, as compared with non-users. The subgroup analysis reported that adolescents were more likely to believe that e-cigarettes are less harmful than tobacco cigarettes, while youth users perceived otherwise. Conclusion: the risk perceptions of e-cigarettes are associated with e-cigarette use among adolescents and youth and could be the focus of health promotion to prevent and curb the uptake of e-cigarettes among young people.

## 1. Introduction

Since the introduction of e-cigarettes, the landscape of tobacco product use among young people, including adolescents, has changed over the past decade [1,2]. Initially introduced as a harm reduction and smoking cessation strategy for adult tobacco smokers, this novel product has gained rapid popularity among adolescents and youth after the entry of JUUL products into the market [3]. Several reasons for its popularity include aggressive targeted marketing towards children and youth, easily accessible products, appealing e-liquid flavours, innovatively intelligent design, high nicotine delivery offered by varied vaping products, and lower risk perceptions [4,5,6]. The data documented by the National Youth Tobacco Survey (NYTS) in the United States (U.S.) reported the rapid increment of e-cigarette use among middle and high school students between 2017 and 2018 in the order of 48% and 78% increases, respectively [7]. In addition, in 2020, an estimated 3.6 million U.S. schoolchildren were currently using e-cigarettes, with e-cigarettes being the most commonly used tobacco product [8,9].

Simultaneously, evidence from prospective cohorts of youth and young adults showed a 46% increase in e-cigarette use in 2018 [10]. A recent retrospective study reported that approximately one-quarter of college students aged 18–25 years in the U.S. either have tried using an e-cigarette at least once or are currently e-cigarette users [11]; this finding is consistent with other studies on the prevalence of e-cigarette use among university students and young adults [12,13,14]. Thus, given that e-cigarette use in this population could potentially ‘renormalise’ tobacco cigarette smoking, the rising prevalence of e-cigarette use among adolescents and youth has become a significant public health concern [15,16]. 

Currently, little evidence is available on the long-term health effects of using e-cigarettes compared to traditional cigarettes. Nevertheless, substantial evidence has shown that short-term health effects contradict the current known perceptions, such as low harm perceptions towards e-cigarettes. Young people who vape were found to have increased respiratory problems such as bronchitis or asthma [17,18], altered brain development [15,19], memory impairment [20,21], risk of cardiovascular problems [22], and nicotine dependence [23,24]. Furthermore, tetrahydrocannabinol (THC)-containing e-cigarettes has been related to e-cigarette or vaping use-associated lung injury (EVALI), which is known as a severe case characterized by chest pain, cough, and shortness of breath, leading to hospitalization and death [25,26]. Approximately 15% and 20% of EVALI cases affected adolescents below 18 years and youth aged 18–20 years in the U.S. and Canada, respectively [27]. Apart from the health effects that considerably outweigh the potential unconfirmed benefits of e-cigarettes as a harm reduction or cessation tool, evidence reveals e-cigarette use could be a gateway to tobacco cigarettes, marijuana, and other illicit drugs [28,29,30].

Concurrently, adolescents and youth were found to have more positive or lower risk perceptions toward e-cigarette use. Around two-thirds of adolescents who preferred e-cigarettes over smoking marijuana perceived e-cigarettes as a healthier alternative [31]. At the same time, college students who were dual users of conventional cigarettes and e-cigarettes reportedly had lower harm perceptions toward e-cigarettes [32]. These perceptions toward e-cigarettes result from e-cigarettes being heavily advertised as less harmful tobacco products than conventional cigarettes and an alternative for cessation [33,34]. In addition, e-cigarette companies employed old marketing strategies, using celebrities, cartoon characters, fashion expos, promotional codes, and loyalty memberships to attract massive support from the youth population [35,36]. As a result, they were more likely to be influenced by e-cigarette advertisements that were reportedly worth remembering, attention-grabbing, thrilling, informative, and convincing [4]. 

Moreover, studies have found that perceptions play a significant role in predicting e-cigarette use among young people. For example, believing that e-cigarettes are less harmful than tobacco was significantly associated with ever using e-cigarettes among adolescents in Great Britain [37]. In addition, college and university students who perceived that e-cigarettes benefited those who quit cigarettes were significantly associated with trying out e-cigarettes [38,39]. More interestingly, the 2015 North Carolina Youth Risk Behavior Survey reported a decrease in the perception of risks of e-cigarettes with each advancing grade [40]. Although studies have reported good associations between perceptions and the use of e-cigarettes among adolescents and youth, different types of perception are significantly related to using e-cigarettes, and findings vary across age groups. For example, U.S. adolescents who perceived e-cigarettes as not addictive were more likely associated with ever using an e-cigarette [41]. Meanwhile, perceiving a lower degree of addictiveness was a predictor of e-cigarette initiation among college students in the U.S [42]. Meanwhile, college students were found to have a positive association between perceived harm reduction and e-cigarette use [39]. 

Despite the increasing prevalence of e-cigarette use among adolescents and youth and numerous primary studies investigating the relationships between perceptions and e-cigarette use, to date no systematic reviews and meta-analyses have been conducted to synthesise the evidence and provide associations between the perceptions and the use of e-cigarettes among adolescents and youth. Consequently, there was a lack of conclusive evidence regarding these issues of interest, which may lead to the ineffective planning and implementation of tobacco policy, along with preventive and health promotion measures. Therefore, this review assessed the association between the perceptions of risks, focusing on the harmfulness and addictiveness of e-cigarettes, and e-cigarette use among adolescents and youth.

## 2. Materials and Methods

The protocol for the present systematic review and meta-analysis was registered with PROSPERO (ID: CRD 42021139995). Therefore, this report complies with the Preferred Reporting Items for Systematic Reviews and Meta-Analyses (PRISMA) statements [43]. 

### 2.1. Eligibility Criteria

This review included primary studies of children (specifically adolescents) and youth participants, aged between 10 and 25 years, with healthcare professionals, pregnant women, and individuals with medical conditions excluded. These individuals were excluded as they may introduce bias in terms of perceptions of e-cigarettes and the use of e-cigarettes influenced by their conditions and occupations. For example, pregnant women may not consume any undesirable harmful substances (i.e., e-cigarettes, tobacco cigarettes), which can increase the risk of having a newborn with a birth defect; therefore, it may reduce the frequency of e-cigarette use that is not attributed to how the participants perceive e-cigarettes [44,45]. Meanwhile, healthcare professionals may own some knowledge regarding e-cigarettes, which could influence their perceptions towards e-cigarettes and the use of e-cigarettes [46,47,48]. The primary studies investigated the association between the perception of the harmfulness and addictiveness of e-cigarettes compared with tobacco cigarettes as the exposure, along with ever using e-cigarettes and including the initiation of e-cigarette use as the outcome of interest in this review. In this review, people who were ever e-cigarette users were defined as those who had tried e-cigarettes, but not in the last 30 days, whereas initiation to using e-cigarettes refers to individuals who had never used e-cigarettes at baseline but self-reportedly used them at least once at follow-up (even one puff or two).

For e-cigarettes being perceived as less or more harmful or addictive than tobacco cigarettes, perceptions that they were equally harmful or addictive was used as the comparator or reference. When the choice of answers to whether e-cigarettes were harmful or addictive was ‘yes’ or ‘no’, the comparator of interest was ‘no’. Meanwhile, if the study measured the perception of the risks with four-point Likert scales (i.e., strongly agree, agree, disagree, strongly disagree) or binary (agree and disagree) answer options, the comparator of interest was ‘disagreed’ (‘strongly disagree’ combined with ‘disagree’). Only observational studies such as cross-sectional, comparative cross-sectional, case-control, nested case-control, retrospective, and prospective cohort studies, including mixed-methods, were eligible for inclusion.

### 2.2. Sources of Data and Search Strategy

The search strategies consisted of a combination of Medical Subject Headings (MeSH) terms, free text, and synonyms deployed after the initial search in PubMed. Individual search strategies were formulated to search for eligible studies from fourteen databases, including PubMed, MEDLINE, Embase, Cochrane Library, CINAHL, Scopus, Web of Science, Science Direct, ProQuest, Dentistry & Oral Sciences Source via EBSCOHost, Psychology & Behavioural Science Collection via EBSCOHost, Google Scholar, NIH Library, and the National Addiction & HIV Data Archive Program from the inception of e-cigarettes until March 2022; the studies were limited to research articles and English language papers. In conjunction with lateral searches of ISI Web of Science, Scopus, and Google Scholar, we also performed manual searches of reference lists of the reviews and included studies. We additionally screened grey literature through Google Scholar. Supplementary Table S1 depicts the details of the PubMed search strategy as an example.

### 2.3. Data Management and Selection of Studies

All identified studies were imported into EndNote 9 (Clarivate, London, United Kingdom), followed by a de-duplication of the records. The selection process started with the titles and abstracts being screened against the eligibility criteria by two independent calibrated reviewers (Kappa = 0.83). Subsequently, the full text of the potentially eligible studies was independently assessed again by the two reviewers (A.S.A. and R.M.) for inclusion in the present review. We resolved discrepancies by consulting a third reviewer (M.J.) and recorded the reasons for the study’s exclusion. In case of duplication, the most extensive studies were selected. Grey literature, Master’s dissertations, or PhD theses were included if they met the eligibility criteria. Review articles were excluded unless we could not find the original primary studies, and a summary of statistical data could be extracted from the review. In addition to the references list of the included studies, we also used the reviews as key sources for further study identification. 

### 2.4. Data Extraction

Two calibrated reviewers (A.S.A. and R.M.) independently extracted the data using the reduced data extraction method by focusing on the aspects critical to the results (i.e., exposure and primary outcomes) and aspects which required more subjective interpretations (i.e., patients characteristics and added outcomes) [49,50]. Data were extracted based on the PICO/S framework; (1) population, (2) exposure, (3) comparator, (4) outcome, and (5) study design. For example, we extracted data on the characteristics of participants, including the age group, perceptions of harmfulness and addictiveness of e-cigarettes, association between perceptions of risks and e-cigarette use, and type of study, authors, year of publication, etc.

### 2.5. Risk of Bias Assessment

The methodological quality of cross-sectional studies was assessed using scores from the Agency for Healthcare Research Quality (AHRQ) to classify studies as high-quality (scores 8–11), moderate-quality (scores 4–7), or low-quality (scores 0–3). The AHRQ evaluation comprised 11 items spread into five domains; selection bias (4 items), performance bias (2 items), attrition bias (1 item), detection bias (2 items), and reporting bias (2 items), with answer options ranging ‘Yes’, ‘No’, ‘Can’t tell’ or ‘Not applicable’. The ‘Yes’ answer was awarded one score point, and the assessment’s overall score ranged from 0 to 11. The methodological quality of the cohort studies was assessed using the Newcastle–Ottawa Scale (NOS), which covered three domains: selection (4 items), comparability (1 item), and outcome (3 items). Each item was awarded a maximum of one star for selection and outcome/exposure and two stars for comparability to categorize studies into high-quality (scores 7–9), moderate-quality (scores 4–6), or low-quality (scores 0–3). 

### 2.6. Quality of Evidence

The Grades of Assessment, Development, and Evaluation (GRADE) system was used to assess the quality of evidence in this review. Each outcome was rated as high, moderate, low, or very low based on the downgrading (risk of bias, inconsistency, indirectness, imprecision, publication bias) and upgrading factors (effect size, dose response, plausible residual confounding). The evidence summaries for each outcome were developed using the GRADEpro GDT tool.

### 2.7. Statistical Analysis

Using RevMan Software (Version 5.3)(Copenhagen, Denmark) for statistical analysis, the odds ratios and their corresponding 95% confidence intervals were calculated by extracting the number of events and totals for each outcome using the Mantel–Haenszel statistical method for dichotomous outcomes between the comparison groups. In the event this method was not used, each effect estimate from individual studies was then gathered using the generic inverse variance method to calculate the pooled effect estimates [49]. A random effect model was employed to calculate the pooled effect estimates when a considerable heterogeneity (I^2^ > 75%) among the included studies was present. A sensitivity analysis was performed to assess the robustness of the pooled estimates. Publication bias was also examined using a funnel plot when a minimum of ten included studies for each outcome was present.

### 2.8. Subgroup Analysis

Two subgroup analyses investigated whether age groups, adolescents, and youth modify the associations between perceived harmfulness and ever being an e-cigarette user in youths; the adolescent group comprised middle and high school students. The youth group was inclusive of university and college students at the time of recruitment. Unfortunately, we did not perform a subgroup analysis for perceived addictiveness because fewer than ten studies were included [49].

## 3. Results

### 3.1. Search Results

**Figure 1:**
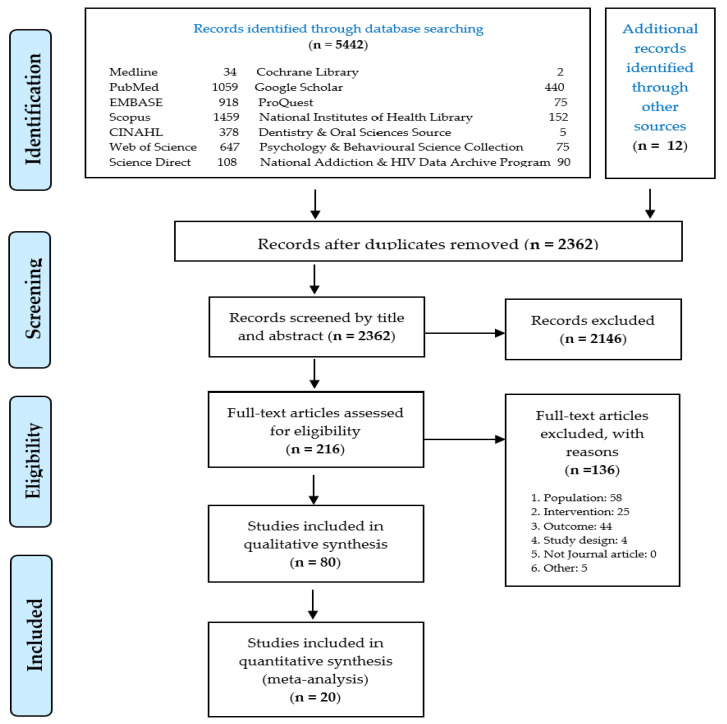
PRISMA flow diagram summarizing the search, identification, and selection of the included studies.

The literature search identified 2362 eligible records after the removal of duplicates. Two hundred and sixteen articles were eligible for full-text screening. After excluding articles according to the established inclusion and exclusion criteria, twenty articles were included in the systematic review and meta-analysis. Figure 1 describes the PRISMA flowchart for the search, identification, and screening of the eligible articles.

### 3.2. Characteristics of the Included Studies

Two included studies were cohort, while the remaining 18 were cross-sectional. These studies included 887,182 adolescents and 10,304 youth. Most of the studies involved both middle (or secondary) and high school students (*n* = 8), followed by either middle, secondary/high school students (*n* = 5), university/college students (*n* = 4), and community/residence (*n* = 3). One half of the included articles were from the United States, while one-fifth originated from Asian countries. Half of the studies started their investigation prior to 2015 when JUUL was successfully introduced into the tobacco market. Most of the studies involved only one risk perception, while one-fifth measured both the perceived harmfulness and addictiveness as their exposure of interest. Table 1 summarizes the characteristics of all included studies for this review and meta-analysis. 

### 3.3. Risk of Bias

**Figure 2:**
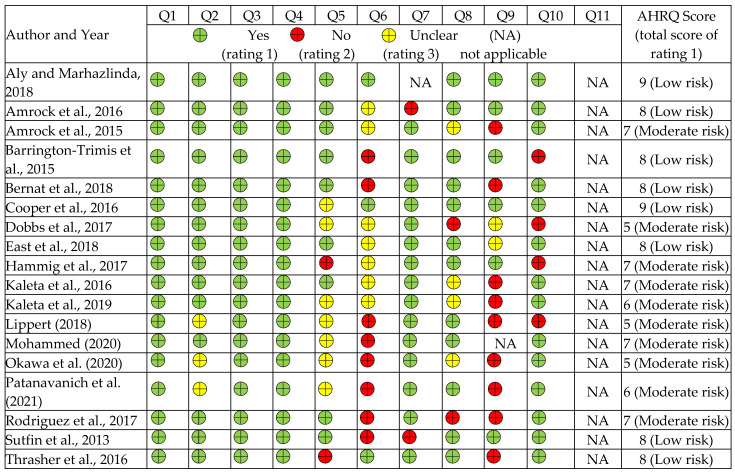
Risk of bias of the cross-sectional studies included in the review using the AHRQ tool [37,51,52,53,55,56,57,58,,59,60,61,62,63,64,65,66,67,68].

**Figure 3:**
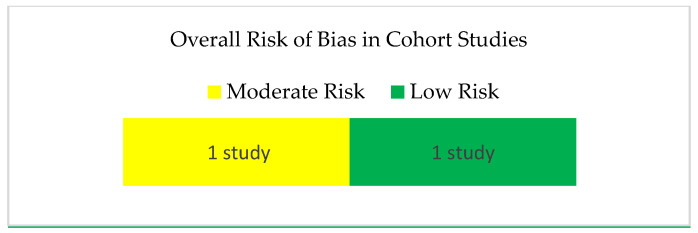
Risk of bias assessment of the cohort studies included in the review using the NOS tool.

Of the 18 studies assessed by the AHRQ instrument, eight had a low risk of bias while ten had a moderate risk (Figure 2, Supplementary Table S2). At the same time, the two cohort studies assessed by the NOS tool were each judged as having a moderate and low risk of bias (Figure 3, Supplementary Table S3).

### 3.4. Perceived Harmfulness of E-Cigarettes and Ever E-Cigarette User

**Figure 4:**
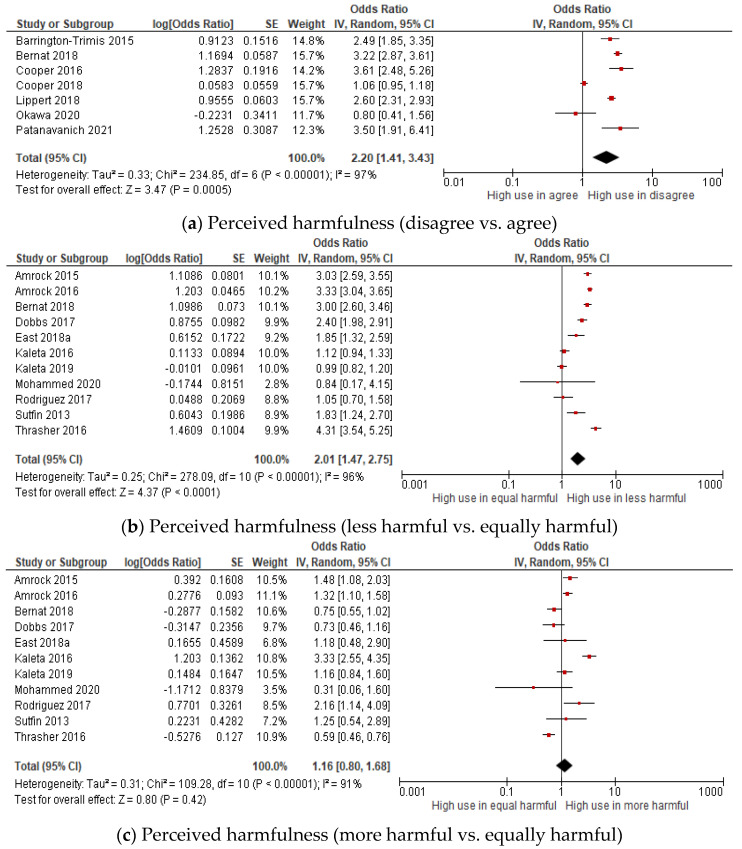

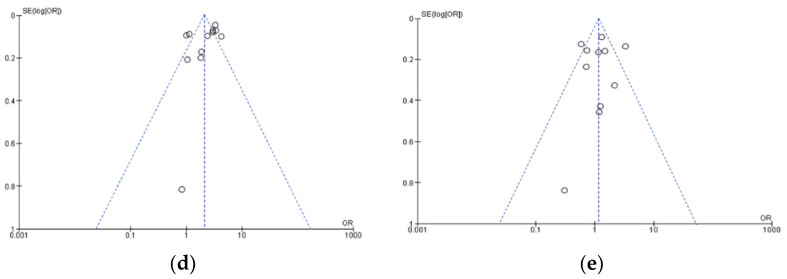
Forest plot of the meta-analysis for perceived harmfulness (**a**–**c**) and ever e-cigarettes use in young people (adolescent and youth) [37,42,53,54,55,56,57,58,60,61,62,63,64,65,66,67,68]. Each study is identified by their first author. The individual effect estimates are identified as odds ratios with lower and upper limits (95% confidence interval). Funnel plots of the meta-analysis of the association between perceived harmfulness and ever e-cigarette use. (**d**) less harmful vs. equally harmful group. (**e**) more harmful vs. equally harmful group.

A total of seventeen studies measured the association between perceived harmfulness and ever e-cigarette use among young people (adolescent and youth) (Figure 4a–c). The pooled estimates of the odds ratio showed that young people who were ever e-cigarette users were two times more likely than the non-users to have disagreed that e-cigarettes are harmful (OR: 2.20, 95% CI: 1.41–3.43) and perceived e-cigarettes as less harmful than tobacco cigarettes (OR: 2.01, 95% CI: 1.47–2.75), revealing a positive association. In other words, the odds of being ever e-cigarette users were significantly higher among those who disagreed that e-cigarettes are harmful and perceived e-cigarettes as less harmful than tobacco cigarettes. No significant association was found between perceptions of more harm and the use of e-cigarettes. Significant substantial heterogeneity was observed between studies in disagree versus agree groups (I^2^ = 97%), i.e., in the less or more harmful vs. equally harmful group (I^2^ = 96%, 91%). Thus, a sensitivity analysis was performed. The publication bias was assessed for the less harmful versus equally harmful group and more harmful versus equally harmful group, showing symmetrical funnel plots and a low-level publication bias (Figure 4d,e).

### 3.5. Perceived Addictiveness of E-Cigarettes and Ever E-Cigarette User

**Figure 5:**
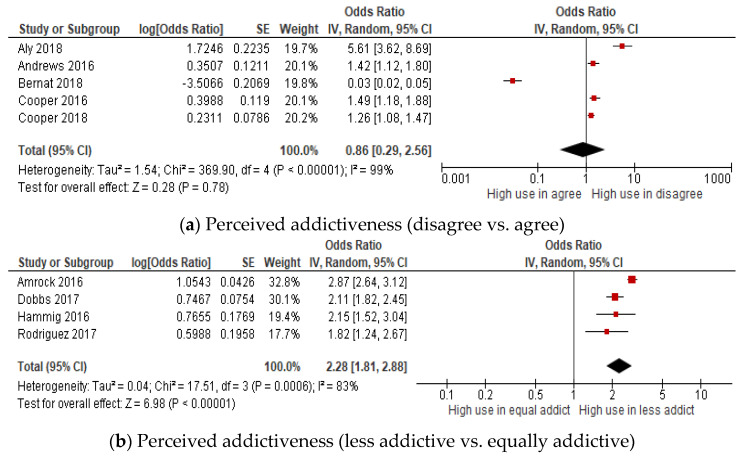

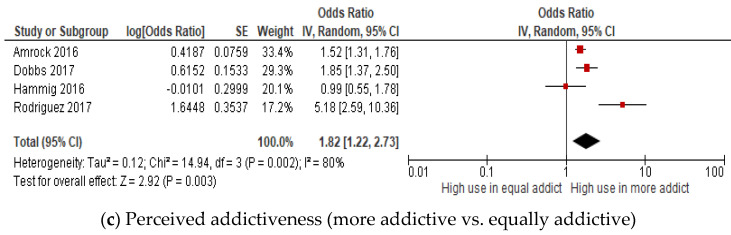
Forest plot of the meta-analysis for perceived harmfulness (**a**–**c**) and ever e-cigarettes use in young people [42,51,52,54,56,57,58,59,66]. Each study is identified by their first author. The individual effect estimates are identified as odds ratios with lower and upper limits (95% confidence interval).

Nine studies measured the association between perceived addictiveness and ever e-cigarette use among adolescents and youth (Figure 5a–c). The results showed that those young people who were ever e-cigarette users were 2.3 and 1.8 times more likely to perceive e-cigarettes as less addictive (OR: 2.28, 95% CI: 1.81–2.88) or perceive e-cigarettes as more addictive (OR: 1.82, 95% CI: 1.22–2.73) than tobacco cigarettes, as compared with the non-users. In other words, the odds of being ever e-cigarette users were significantly higher among those who perceived e-cigarettes as less or more addictive than tobacco cigarettes. Significant heterogeneity was visually observed in the disagree vs. agree group (I^2^ = 99%), in the less addictive vs. equally addictive group (I^2^ = 83%) and in the more addictive vs. equally addictive group (I^2^ = 80%). No publication bias was assessed due to the limited number of included studies.

### 3.6. Sensitivity Analysis

The findings of the sensitivity analysis showed that the pooled estimates for the association between the perceived harmfulness and addictiveness and the ever e-cigarette users changed after removing the included studies one-by-one (Table 2). For example, the removal of Cooper et al. (2018) in the disagree vs. agree group and Kaleta et al. (2016) in the less harmful vs. equally harmful group in the meta-analysis of the association between perceived harmfulness and ever using e-cigarettes significantly increased the pooled estimates and reduced the heterogeneity (OR = 2.68, 95% CI 2.16–3.34, I^2^ = 79% in disagree vs. agree group; OR = 2.16, 95% CI 1.62–2.90, I^2^ = 95% in the less harmful vs. equally harmful group, respectively). For the association between perceived addictiveness and ever-users, after removing Bernat et al. (2018) in the disagree vs. agree group, the pooled estimates increased and had their heterogeneity reduced (OR = 1.88, 95% CI 1.21–2.90, I^2^ = 92%). However, removing Amrock et al. (2016) in the less addictive vs. equally addictive group and Rodriguez et al. (2017) in the more addictive vs. equally addictive group only significantly reduced the heterogeneity (OR = 2.08, 95% CI 1.83–2.37, I^2^ = 0% in the less addictive vs. equally addictive group and OR = 1.53, 95% CI 1.22–1.93, I^2^ = 45% in the more addictive vs. equally addictive group, respectively) (Table 2, Supplementary Figure S1).

### 3.7. Subgroup Analysis

**Figure 6:**
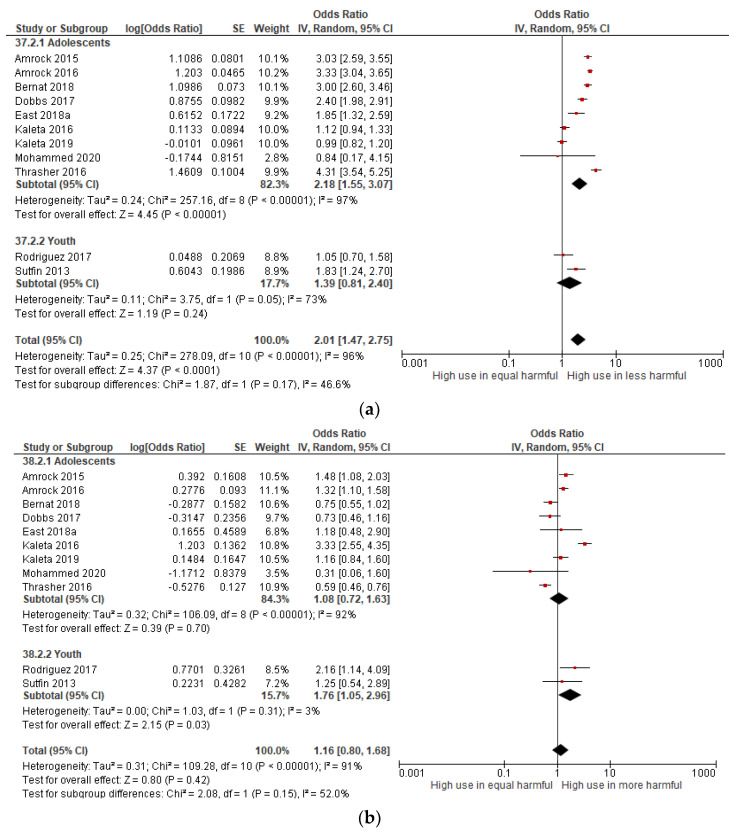
Forest plot showing the age group effects for perceived harmfulness and ever e-cigarette use in young people according to age groups [37,52,53,56,58,60,61,63,66,67,68]. (**a**) Less harmful versus equally harmful group and (**b**) more harmful versus equally harmful group.

A subgroup analysis according to age group was performed on the eleven included studies for the perceived harmfulness and ever e-cigarette use (Figure 6). Although there was no statistically significant between subgroups (*p* > 0.1), which indicated no effect of age groups on the associations, there was a significant association between perceived harmfulness and ever-users within the subgroups. The pooled effect estimates show that in comparison to the non-user, adolescent ever e-cigarette users were significantly positively associated with perceiving e-cigarettes as less harmful than tobacco cigarettes (OR: 2.18, 95% CI: 1.55–3.07, I^2^ = 97%); however, the association was not significant in the youth group. On the other hand, compared to the non-users, those youth ever e-cigarette users were significantly associated with perceiving e-cigarette as more harmful than tobacco cigarettes (OR: 1.76, 95% CI: 1.05–2.96, I^2^ = 3%); however, the association was not significant among adolescents ever-users. The non-significant subgroup effect of age groups may have been attributed to the uneven studies distribution and participants within both subgroups, adolescents, and young adults.

### 3.8. Quality of Evidence

Based on the GRADE ratings, the quality of evidence in this current review ranges from very low (40%) to low-quality (60%). For both associations between harm and addictive perception and ever e-cigarette user among young people, an equal number of low-quality (3) and very low-quality (3) evidence were found. For example, two instances of low-quality evidence included the disagree vs. agree group and less harmful vs. equally harmful group in the meta-analysis of the association between perceived harmfulness and ever-users. At the same time, another instance of low-quality evidence was the less addictive vs. equally addictive group for the association between perceived addictiveness and ever-users. The remaining three very low-quality pieces of evidence included the perceived harmfulness (more harmful vs. equally harmful group) and perceived addictiveness (disagree vs. agree group and more addictive vs. equally addictive group), respectively. For subgroup analysis by age groups, the quality of evidence ranges from low (75%) and very low (25%). Supplementary Table S4 summarizes the quality of the body of evidence.

## 4. Discussion

To our knowledge, this is the first meta-analysis focused on the association between perceived harm and addiction to e-cigarettes and their use among young people consisting of adolescents and youth. One of our main findings revealed that ever e-cigarette users were two times more likely than non-users to have disagreed that e-cigarettes are harmful and to perceive e-cigarettes as less harmful than tobacco cigarettes. The findings are consistent with previous primary studies that demonstrated a positive association between perceived harmfulness and ever e-cigarette use in young people [55,56,57,62,65]. For example, those middle and high school students who disagreed that e-cigarettes are harmful were 2.6 to 3.6 times more likely to have tried e-cigarettes at least once than those who agreed [57,62]. Similarly, ever e-cigarette users among young people in North Carolina were significantly related to a lower harm perception of e-cigarettes [67]. However, other studies reported no significant association between harmful perception and ever using e-cigarettes among young people [42,60,63,64]. 

Several reasons could explain how the perceptions of lower harmfulness of e-cigarettes may be related to the use of e-cigarettes among young people. First, young individuals, particularly adolescents, lack knowledge of the harmful aspect of e-cigarette constituents, regardless of their high-level awareness of e-cigarettes [69,70,71]. The sources of understanding mostly consisted of mass media, the internet, and advertisements, which provide mixed messages to viewers [72]. The presence of harmful constituents such as nanoparticles, heavy metals (i.e., aluminium, copper, magnesium), formaldehyde, and vitamin E acetate has been shown to cause damage to the human lungs [73,74,75]. The biggest outbreak of e-cigarette health injury is, by far, the EVALI epidemic in 2019 [25]. As a result of the lack of knowledge, most young individuals may perceive e-cigarettes as less harmful than tobacco cigarettes, leading to a higher likelihood of using e-cigarettes. Secondly, e-cigarettes have been reportedly as being widely perceived as less harmful than tobacco cigarettes, particularly due to the appealing flavoured e-cigarettes [76,77,78,79]. Moreover, the misconception was partially due to health claims strongly suggested by manufacturers or conclusions made by health experts or medical organizations. Researchers who related to the tobacco industry or were funded by tobacco companies were less likely to report the harmful effects of using e-cigarettes [80]. In this present review, five included studies were conducted without funding closure; thus, the impact of tobacco company bias was non-conclusive. Moreover, e-cigarettes have been inferred to be almost 95% safer than conventional cigarettes [81]. Additionally, e-cigarettes are considered a ‘safe’ alternative to tobacco cigarettes. Lower perceived harm from e-cigarettes has been found to predict the initiation of e-cigarettes among non-smokers and non-current tobacco smokers among youth [42,77]. In addition, e-cigarettes are perceived as less harmful than tobacco cigarettes due to their lack of combustion [82,83]. Their tobacco combustion causes the overwhelming harm of tobacco cigarettes and, naturally, other modes of tobacco use are less dangerous than combustible products. Therefore, this reasoning promotes e-cigarette use among individuals with some respiratory problems (i.e., asthma, chronic obstructive pulmonary disease (COPD), pulmonary fibrosis) [84].

Another main finding revealed that young ever e-cigarette users were 2.3 times more likely than the non-users to perceive e-cigarettes as less addictive than tobacco cigarettes while also being 1.8 times more likely to perceive e-cigarettes as more addictive, showing a positive association. However, the effect size of the association was more substantial for less perceived addictiveness. Our findings that the ever-users were more likely to have perceived e-cigarettes as less addictive compared with non-users concurs with other studies, wherein the young individuals who perceived e-cigarettes as less addictive than tobacco cigarettes were more likely to have ever used e-cigarettes [52,58,59,66]. Another study also found lifetime teen e-cigarette users had two-fold increases than the non-users in perceiving e-cigarettes as less addictive and more addictive than tobacco cigarettes [58]. However, our findings that young ever e-cigarette users were more likely to perceive e-cigarettes as more addictive than the non-users were unsupported by other studies, which found that greater addictive perceptions are not significantly associated with reduced odds of e-cigarette usage among young people [59]. 

Several factors could explain the less addictive and/or more addictive perception of e-cigarettes being related to ever e-cigarette use in the youths. To begin with, this would depend on the types, models, and generations of e-cigarettes used. In particular, the more advanced e-cigarette devices, i.e., third generation and above, can produce a larger volume of aerosol and higher nicotine content with a high-capacity battery of varying strength [85]. Therefore, those young people who may have regularly used e-cigarettes producing a higher dose of nicotine per inhalation may have perceived e-cigarettes as more addictive than tobacco cigarettes. However, not all brand e-cigarettes in the market contain high nicotine levels [17], promoting higher and regular e-cigarette uptake, which could explain why these young people also have perceived e-cigarettes as less addictive than tobacco cigarettes. In addition, a higher level of nicotine dependence was observed when using e-cigarettes compared with tobacco smoking [85]. The level of nicotine dependence as measured by the Fagerström Test for Nicotine Dependence (FTND) were evaluated to be more than double when using e-cigarettes (mean of 3.5) as compared with smoking tobacco cigarettes. This may have led e-cigarette users to believe e-cigarettes are more addictive than conventional cigarettes. Another salient point is the user experience (user vs. non-user). Experimental users may perceive e-cigarettes as less addictive than regular users due to no experience or a lack of experience with the taste of nicotine [71]. Finally, our review included studies conducted from 2012 to 2016 with changes in addictive perception among young people over the years. This was proven by an analysis of the National Youth Tobacco Survey (NYTS), which found an increase of 360% in the perceived addictiveness, changing from 7.3% in 2016 to 26.3% in 2019 [86].

Statistically, our review found that age groups do not significantly modify the associations between perceived harmfulness and ever e-cigarette use among young people. The subgroup analysis also did not reduce the heterogeneity, indicating the age groups (adolescents and young adults) do not explain the heterogeneity. However, the uneven covariate distribution where many more studies were among adolescents than in the youth group could be why the analysis did not statistically detect the subgroup effects that were descriptively present. Therefore, although the subgroups test was not statistically significant, we observed a significant positive association within the adolescents and youth groups. Interestingly, in comparison to the non-users: (1) the adolescent ever-users were significantly two times more likely to have perceived e-cigarettes as less harmful than tobacco cigarettes; and (2) the young adult ever-users were significantly 1.7 times more likely to have perceived e-cigarettes as more harmful than tobacco cigarettes. This suggests that while both groups’ perceptions of harmfulness were positively associated with e-cigarette use, there was a difference in how adolescents and youths perceived e-cigarettes compared to tobacco cigarettes. While the adolescents were inclined to believe that e-cigarettes were less harmful than tobacco cigarettes, the young adults felt the opposite. These results portray a more complex association between perceived harmfulness and e-cigarette use in young people, which may not be explained by age alone.

The results of our review align with the current perspective of an increasing trend in lower harm perception of e-cigarettes among adolescents. According to the 2012 National Youth Tobacco Survey (NYTS) data, approximately a third of U.S. middle and high school students believed that e-cigarettes are less harmful than tobacco cigarettes [87]. Subsequently, the lower harm perception among school students had increased more than two-fold based on the 2014 NYTS data analysis [52]. Furthermore, at least one-third of the youth population thought that e-cigarettes are less harmful than tobacco cigarettes because the e-cigarettes’ aerosol is seen as ‘flavoured smoke’ [88]. Simultaneously, adolescents also showed a significant upsurge in e-cigarette use between 2017 and 2018. The most used tobacco product device among middle and high school students in 2017 was e-cigarette products (3.3%; 0.39 million vs. 11.7%; 1.73 million), indicating that the use of this product was even higher in younger youth. The rapid upsurge of e-cigarette use among adolescents was attributed to the popularity and growth of JUUL products. Indeed, JUUL is associated with the e-cigarette epidemic among young people, attracting non-e-cigarette users and accelerating the usage among current users through appealing flavours and highly addictive nicotine content [89]. The concomitant increasing trend of lower harm perception and e-cigarette use among young individuals may explain how a less harmful perception of e-cigarettes among young people is positively associated with e-cigarette use, as revealed in this current review. Furthermore, e-cigarette advertisement has heavily targeted adolescents; these include billboards advertisements, product packaging, and a wide distribution of social media advertisements. More than two-thirds (78%) of middle and high school students were exposed to at least one e-cigarette ad [90]. Pod-type e-cigarettes such as JUUL come in fun and attractive packaging, with USB-like form, pens, or inhalers. This can be personalized according to the customer’s request, similar to a mobile phone case. The varieties of flavourings are exciting to adolescents (i.e., mint, fruity, gummi bear, frosted sugar cookies). Alarmingly, flavourings have been confirmed as one of the risk factors for adolescents attempting to experiment with e-cigarettes [34].

On the other hand, in the perceived more harmful group, a greater likelihood of youth using e-cigarettes have perceived e-cigarettes as more harmful than tobacco cigarettes. A previous study found that North Carolina students who were e-cigarette users were less likely to believe that the use of e-cigarettes was causing great risk. However, the association decreased as students’ grade levels increased [40]. This suggests that as adolescents enter the youth age group, the lower harm perception decreases and the perception of e-cigarettes as more harmful increases. Youths were likely more exposed to negative news about e-cigarettes, which may have influenced their beliefs towards the harmfulness of e-cigarettes. Those exposed to mostly negative e-cigarette news were significantly more likely to increase their beliefs about e-cigarette harms compared with exposure to only positive news headlines [91,92]. Simultaneously, youths could easily access the more advanced e-cigarettes (third generation and above), which are more effective and produce higher addictive nicotine, which leads to the use of e-cigarettes after experimenting despite believing that it is more harmful than tobacco cigarettes [85]. The youth in the included studies of this review were from countries (i.e., Australia, the U.S, and Spain) with independent-thinking societies. Western countries have individualist societies that are unique, independent, and openly express topics [93]. Meanwhile, countries in Asia are more of a collectivist society, where they conform to specific values, loyalties, and traditions [94]. The inclusion of independent thinking may have influenced the youth to perceive e-cigarettes as more harmful yet still allow for their open use, expressing their needs. Although there is no clear evidence to connect independent thinkers and the perception of e-cigarettes as more harmful than tobacco cigarettes, this area can be further explored using a qualitative study design. Additionally, the youth sample mostly comprised dual users who were established smokers and experimental/established e-cigarette users. Previous experience with smoking cigarettes and currently experimenting with e-cigarettes may have caused these youth to believe that e-cigarettes are more harmful than tobacco cigarettes. This may explain why some youths were against regularly using e-cigarettes, as they thought that e-cigarettes were dangerous due to their long-term health effects [95]. 

The present review also observed that the effect size is greater in adolescents, indicating that adolescent users have higher chances of experimenting with e-cigarettes than non-users; the young adult users have a more harmful perception of e-cigarettes. This proposes that among young people, adolescents have a higher probability of trying e-cigarettes when having a lower harm perception of e-cigarettes. The difference in the likelihood of using e-cigarettes in young people is closely related to the vulnerability of adolescents who are more receptive to exposure to e-cigarette marketing advertisements. E-cigarette companies intensely target adolescents via multiple channels, including online platforms and forums. Aside from this channel, social influence, including peer influence and peer pressure to have a sense of belonging among these vulnerable adolescents, is also associated with a higher rate of e-cigarette use.

In summary, limited studies conducted among the youth were observed, indicating that the association between perceived harmfulness and e-cigarette use by age group is not well-established; thus, there is insufficient evidence presented to conclude these findings. Furthermore, the non-significant relationship may be attributed to multifactorial risk factors in both the perceptions of e-cigarettes and the uptake among adolescents and young adults. Therefore, the age group alone may not be able to explain the association between the perceived harmfulness and the lifetime e-cigarette use.

This study has its strength and limitations. First, to the best of our knowledge this review is the first systematic review and meta-analysis to quantify the association between the perceived harm and addiction and the e-cigarette use among adolescents and young adults. Second, this study involved a comprehensive literature search by scanning fourteen available databases, including more restrictive databases such as EMBASE, which is a European-oriented database. Third, the systematic review and meta-analysis in this study employed a standard methodology according to the suggestions made by the Cochrane Collaboration. However, this study also has several limitations. Firstly, high heterogeneity was observed due to the wide age range of the population, which caused varied findings among the individual studies; however, a subgroup analysis was performed to identify the source of heterogeneity. Secondly, most of the included studies for meta-analysis were cross-sectional studies with an inherent risk of bias, including oversampled population and lack of confounders adjustment either through study design or statistics. Thirdly, many studies did not meet the pre-determined eligibility criteria and were thus excluded from the meta-analysis, which may have weakened the quality of the body of evidence. Fourthly, only English-language articles were retrieved; therefore, studies published in other languages may have been ignored, which may have influenced the review’s findings. Finally, majority of the included studies were concentrated in the United States and a few originated from other countries and regions such as Australia, Hong Kong, Malaysia, Mexico, United Kingdom, and Poland. Therefore, the review findings might not represent the overall association trend, as more evidence from other countries is needed to understand the evolving e-cigarette landscape.

## 5. Conclusions

In conclusion, the present review and meta-analysis provide very low to low-quality evidence for harmful and addictive perceptions being positively associated with ever e-cigarette usage. However, adolescent ever-users are positively associated with perceiving e-cigarettes as less harmful. In contrast, the ever-users among the youth were significantly associated with perceiving e-cigarettes as more harmful than tobacco cigarettes. Therefore, based on the evidence, public health policies and health prevention programmes should be carefully planned to curb e-cigarette uptakes among adolescents and young adults by tackling their perception and usage of e-cigarettes. 

## Figures and Tables

**Table 1:** Characteristics of the included studies.

Reference	Country	Study Period	Study Design	Settings	Age	Population	Perception	E-Cigarette Use	Comparators	Outcome	Risk of Bias
										Ever Use	
Aly and Marhazlinda (2018) [51]	Malaysia	2018	CS	University	Mean of 22.5 ± 1.09 years	1475	Addictiveness	Ever use	Disagree vs. agree	Disagree = 43/166, Agree = 57/971 OR = 5.61, 95% CI = 3.62–8.69	Low
Amrock et al. (2016) [52]	United States	2012 and 2014	Repeated CS	Middle and high school	11–18 years	24,658 (2012), 22,007 (2014)	Relative harm Addictiveness	Ever use	Less vs. Equally harmful	Less harmful = 4272/17449, Equally harmful = 603/6790 OR = 3.33, 95% CI = 3.04–3.64	Low
									More vs. Equally harmful	More harmful = 171/1498, Equally harmful = 603/6790 OR = 1.32, 95% CI = 1.10–1.58	
									Less vs. Equally addictive	Less addictive = 2382/6505, Equally addictive = 999/5968 OR = 2.87, 95% CI = 2.64–3.13	
									More vs. Equally addictive	More addictive = 290/1241, Equally addictive = 999/5968 OR = 1.52, 95% CI = 1.31–1.76	
Amrock et al. (2015) [53]	United States	2012	CS	Middle and high school	11–18 years	24,658	Harm of e-cigarettes	Ever use	Less vs. Equally harmful	Less harmful = 1074/7120, Equally harmful = 201/3625 OR = 3.03, 95% CI = 2.59–3.54	Moderate
									More vs. Equally harmful	More harmful = 53/663, Equally harmful = 201/3625 OR = 1.48, 95% CI= 1.08–2.03	
Andrews et al. (2016) [54]	United States	2013–2014	Cohort	Community	Average of 23.52 years	1075	Risk of Addiction	Ever use	Disagree vs. agree	OR = 1.42, 95% CI = 1.12–1.79	Moderate
Barrington-Trimis et al. (2015) [55]	United States	2014	CS from a Cohort study	High school	Mean of 17.3 + 0.6 years	2084	Harm	Ever use	Disagree vs. agree	Disagree = 76/291, Agree = 221/1779 OR = 2.49, 95% CI = 1.85–3.35	Low
Bernat et al. (2018) [56]	United State	2016	CS	High school	14–17 years	22,884	Harm Addictiveness	Ever use	Disagree vs. agree	Disagree = 783/4829, Agree = 561/9886 OR = 3.22, 95% CI = 2.87–3.61	Low
									Less vs. Equally harmful	Less harmful = 1452/9863, Equally harmful = 235/4325 OR = 3.00, 95% CI = 2.60–3.47	
									More vs. Equally harmful	More harmful = 50/1213, Equally harmful = 235/4325 OR = 0.75, 95% CI = 0.55–1.02	
									Disagree vs. agree	Disagree = 637/5126, Agree = 1191/1407 OR = 0.03, 95% CI = 0.02–0.03	
Cooper et al. (2016) [57]	United States	2014–2015	CS	Middle and high school	6th, 8th, 10th grade	3704	Harm Addictiveness	Ever use	Disagree vs. agree	OR = 3.61, 95% CI = 2.48–5.28 OR = 1.49, 95% CI = 1.18–1.87	Low
Cooper et al. (2018) [42]	United States	2014–2017	Cohort	College	18–25 years	2565	Harm Addictiveness	EC Initiator	Disagree vs. agree	OR = 1.06, 95% CI = 0.95–1.18 OR = 1.26, 95% CI = 1.08–1.46	Low
Dobbs et al. (2017) [58]	United States	2014	CS	Middle and high school	9–19 years	27,294	Harm Addictiveness	Ever use	Less vs. Equally harmful More vs. Equally harmful	OR= 2.40, 95% CI = 1.98–2.91 OR = 0.73, 95% CI = 0.46–1.16	Moderate
									Less vs. Equally addictive More vs. Equally addictive	OR= 2.11, 95% CI = 1.82–2.45 OR = 1.85, 95% CI = 1.37–2.49	
East et al. (2018) [37]	Great Britain	2016	CS	Online residence	11–18 years	2103	Relative harm	Ever use	Less vs. Equally harmful	Less harmful = 211/1331, Equally harmful = 45/488 OR = 1.85, 95% CI = 1.32–2.61	Low
									More vs. Equally harmful	More harmful = 6/56, Equally harmful = 45/488 OR = 1.18, 95% CI = 0.48–2.91	
Hammig et al. (2017) [59]	United States	2014	CS	Middle and high school	6th-12th grade	736,158	Addictiveness	E-cigarettes initiator	Less vs. Equally addictive More vs. Equally addictive	OR = 2.15, 95% CI = 1.52–3.02 OR = 0.99, 95% CI = 0.55–1.78	Moderate
Kaleta et al. (2019) [60]	Poland	2017–2018	CS	Secondary and high school	13–19 years	1693	Relative harm	Ever use	Less vs. Equally harmful	Less harmful = 318/1256, Equally harmful = 254/994 OR = 0.99, 95% CI = 0.82–1.20	Moderate
									More vs. Equally harmful	More harmful = 63/221, Equally harmful = 254/995 OR = 1.16, 95% CI = 0.84–1.60	
Kaleta et al. (2016) [61]	Poland	2014–2015	CS	Secondary and high school	13–19 years	3552	Harm	Ever use	Less vs. Equally harmful	Less harmful = 384/1875, Equally harmful = 260/1387 OR = 1.12, 95% CI = 0.94–1.33	Moderate
									More vs. Equally harmful	More harmful = 126/290, Equally harmful = 260/1387 OR = 3.33, 95% CI = 2.55–4.35	
Lippert (2018) [62]	United States	2014	CS	Middle and high school	6th–12th grade	22,007	Harm	Ever use	Disagree vs. Agree	OR = 2.60, 95% CI = 2.31–2.92	Moderate
Mohammed (2020) [63]	Malaysia	2020	CS	Secondary school	13–14 years	240	Harm	Ever use	Less vs. Equally harmful	Less harmful = 2/32, Equally harmful = 9/123 OR = 0.84, 95% CI = 0.17, 4.15	Moderate
									More vs. Equally harmful	More harmful = 2/85, Equally harmful = 9/123 OR = 0.31, 95% CI = 0.06, 1.60	
Okawa et al. (2020) [64]	Japan	2019	CS	Online community	15–19 years	2414	Harm of EC	Ever use	Disagree vs. Agree	AOR = 0.80, 95% CI = 0.41–1.57	Moderate
Patanavanich et al. (2021) [65]	Thailand	2019	CS	Secondary school	Average of 19 years	6238	Harm	Ever use	Disagree vs. Agree	AOR = 3.51, 95% CI = 1.92–6.41	Moderate
Rodriguez et al. (2017) [66]	Spain	2015–2016	CS	University	Mean of 21.9 ± 3.9 years	745	Harmfulness Addiction	Ever use	Less vs. Equally harmful	Less harmful = 81/364, Equally harmful = 46/215 OR = 1.05, 95% CI = 0.70, 1.58	Moderate
									More vs. Equally harmful	More harmful = 20/54, Equally harmful = 46/215 OR = 2.16, 95% CI = 1.14, 4.09	
									Less vs. Equally addictive	Less addictive = 74/264, Equally addictive = 62/351 OR = 1.82, 95% CI = 1.24, 2.67	
									More vs. Equally addictive	More addictive = 20/38, Equally addictive = 62/351 OR = 5.18, 95% CI = 2.59, 10.36	
Sutfin et al. (2013) [67]	United States	2009	CS	University	Mean of 20.5 + 2.9 years	4444	Harm	Ever use	Less vs. Equally harmful	Less harmful = 97/1042, Equally harmful = 37/697 OR = 1.83, 95% CI = 1.24, 2.70	Low
									More vs. Equally harmful	More harmful = 7/107, Equally harmful = 37/697 OR = 1.25, 95% CI = 0.54, 2.89	
Thrasher et al. (2016) [68]	Mexico	2015	CS	Middle school	12–13 years	10,146	Relative harm	Ever use	Less vs. Equally harmful	Less harmful = 550/2011, Equally harmful = 142/1769 OR = 4.31, 95% CI = 3.54, 5.25	Low
									More vs. Equally harmful	More harmful = 115/2333, Equally harmful = 142/1769 OR = 0.59, 95% CI = 0.46, 0.76	

**Table 2:** Pooled odds ratio and 95% confidence interval (CI) after sensitivity analysis.

Perceptions	Comparators	No. of Studies	Pooled Odds Ratio	95% CI	I^2^	*p*-Value	Supplementary
Harmfulness	Disagree vs. Agree	7	2.68	2.16–3.34	79%	<0.000	Figure S1a
	Less harmful vs. Equally harmful	11	2.16	1.62–2.90	95%	<0.000	Figure S1b
	More harmful vs. Equally harmful	11	1.03	0.77–1.36	80%	0.85	Figure S1c
Addictiveness	Disagree vs. Agree	5	1.88	1.21–2.90	92%	0.005	Figure S1d
	Less addictive vs. Equally addictive	4	2.08	1.83–2.37	0%	<0.000	Figure S1e
	More addictive vs. Equally addictive	4	1.53	1.22–1.93	45%	0.0002	Figure S1f

## Data Availability

Not applicable.

## Supplementary Materials

The following supporting information can be downloaded at: https://www.mdpi.com/article/10.3390/children9111678/s1, Figure S1: Forest plot of the meta-analysis for perceived risks, (a–c) perceived harmfulness, (d–f) perceived addictiveness and ever e-cigarettes use in young people after sensitivity analysis. Each study is identified by their first author. The individual effect estimates is identified as odds ratios with lower and upper limits (95% confidence interval); Table S1: Details of PubMed search strategy; Table S2: Risk of bias of cross-sectional studies based on AHRQ tool; Table S3: Risk of bias of cohort studies based on NOS tool. Table S4: Quality assessment of the present review (GRADE Evidence Profile).
