# Supplementary material for: Association between Harmful and Addictive Perceptions of E-Cigarettes and E-Cigarette Use among Adolescents and Youth—A Systematic Review and Meta-Analysis
Source: Children (Basel). 2022 Oct 31;9(11):1678. doi: 10.3390/children9111678 (PMC9689130; doi:10.3390/children9111678)
Supplement: Supplementary file 1 [file children-09-01678-s001.zip › children-1928190-supplementary.pdf]

children MDPI

Supplementary Materials

Supplementary Table S1. Details of PubMed search strategy.

Focus	Operator	Search Terms
#1 Youth	Keywords	((((((((((((((((((("young adult"[MeSH Terms]) OR adolescent[MeSH Terms]) OR adolescence[MeSH Terms]) OR "Secondary School"[MeSH Terms]) OR "young adult*"[Title/Abstract]) OR youth*[Title/Abstract]) OR adolesc*[Title/Abstract]) OR teen*[Title/Abstract] OR "Secondary School"[Title/Abstract]) OR "Secondary School*"[Title/Abstract]) OR "Secondary School Children"[Title/Abstract]) OR "middle school children"[Title/Abstract]) OR "high school children"[Title/Abstract]) OR "college student*"[Title/Abstract]) OR "university student*"[Title/Abstract])
#2 Perception	Keywords	(((((((((((((((((((("perception"[MeSH Terms]) OR "thinking"[MeSH Terms]) OR "critical thinking"[MeSH Terms]) OR perceiv*[Title/Abstract]) OR perception*[Title/Abstract]) OR perspective[Title/Abstract]) OR opinion*[Title/Abstract]) OR view[Title/Abstract]) OR viewpoint*[Title/Abstract]) OR thinking*[Title/Abstract]) OR "thinking skill*"[Title/Abstract]) OR thought*[Title/Abstract]) OR "critical thinking*"[Title/Abstract]) OR impression*[Title/Abstract]) OR attitude[Title/Abstract])
#3 E-cigarettes	Keywords	((((((((((((((((((("electronic nicotine delivery systems"[MeSH Terms]) OR e-cigarettes[MeSH Terms]) OR "cigarettes, electronic"[MeSH Terms]) OR vaping[MeSH Terms]) OR "electronic cigarette*"[Title/Abstract]) OR "electronic nicotine delivery systems"[Title/Abstract]) OR ENDS[Title/Abstract]) OR e-cigarette*[Title/Abstract]) OR "e cigarette*"[Title/Abstract]) OR e-cig*[Title/Abstract]) OR "e cig*"[Title/Abstract]) OR vap*[Title/Abstract]) OR "E-Cigarette Use*"[Title/Abstract]) OR "E Cigarette Use*"[Title/Abstract]) OR "E-Cig Use*"[Title/Abstract]) OR "E Cig Use*"[Title/Abstract])
#4	Boolean Operator	#1 AND #2 AND #3
#5	Limits	#4 Filters activated: Humans, English, Child: birth-18 years, Adult: 19+ years, Adult: 19-44 years, Child: 6-12 years, Young Adult: 19-24 years, Adolescent: 13-18 years

Supplementary Table S2 Risk of bias of cross-sectional studies based on AHRQ tool

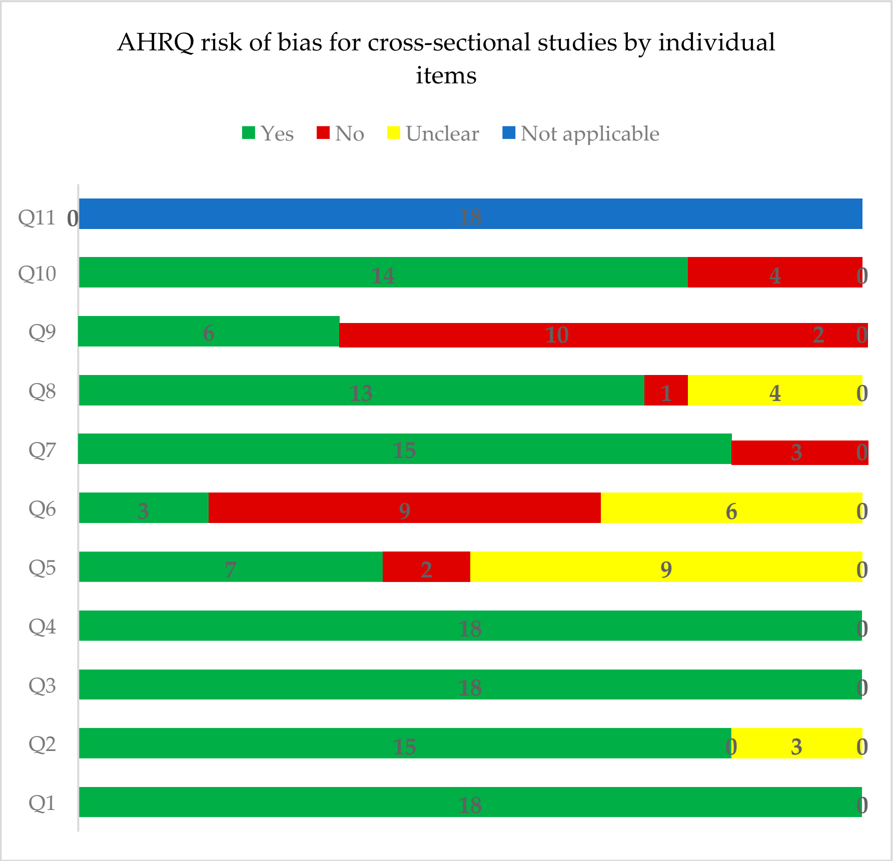

Note: Selection bias (Q1-Q4), performance bias (Q5, Q6), attrition bias (Q7), detection bias (Q8, Q9) and reporting bias (Q10, Q11)

Supplementary Table S3 Risk of bias of cohort studies based on NOS tool

Study ID	Representative-ness of the cohort	Selection of the non-exposed cohort	Ascertain-ment of exposure	Outcome of interest was not present at start	Compa-rability*	Assess-ment of outcome	Follow-up long enough for outcomes	Adequacy of follow up	Score	Quality
Andrews et al., 2016	*	*	-	-	-	-	*	*	4	Moderate
Cooper et al., 2018	*	*	-	*	*	-	*	*	7	High

(a) Perceived harmfulness (disagree vs agree)

Study or Subgroup	log[Odds Ratio]	SE	Weight	Odds Ratio IV, Random, 95% CI
Barrington-Trimis 2015	0.9123	0.1516	18.1%	2.49 [1.85, 3.35]
Bernat 2018	1.1694	0.0587	25.3%	3.22 [2.87, 3.61]
Cooper 2016	1.2837	0.1916	15.0%	3.61 [2.48, 5.26]
Cooper 2018	0.0583	0.0559	0.0%	1.06 [0.95, 1.18]
Lippert 2018	0.9555	0.0603	25.2%	2.60 [2.31, 2.93]
Okawa 2020	-0.2231	0.3411	7.6%	0.80 [0.41, 1.56]
Patanavanich 2021	1.2528	0.3087	8.7%	3.50 [1.91, 6.41]
**Total (95% CI)**			**100.0%**	**2.68 [2.16, 3.34]**

Heterogeneity: Tau² = 0.04; Chi² = 23.27, df = 5 (P = 0.0003); I² = 79%
Test for overall effect: Z = 8.92 (P < 0.00001)

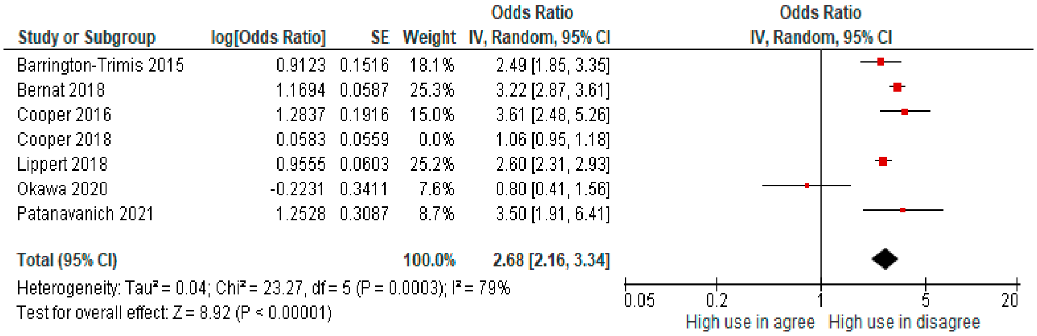

(b) Perceived harmfulness (less harmful vs equal harmful)

Study or Subgroup	log[Odds Ratio]	SE	Weight	Odds Ratio IV, Random, 95% CI
Amrock 2015	1.203	0.0465	11.6%	3.33 [3.04, 3.65]
Amrock 2016	1.1086	0.0801	11.4%	3.03 [2.59, 3.55]
Bernat 2018	1.0986	0.073	11.4%	3.00 [2.60, 3.46]
Dobbs 2017	0.8755	0.0982	11.2%	2.40 [1.98, 2.91]
East 2018	0.6152	0.1722	10.2%	1.85 [1.32, 2.59]
Kaleta 2016	0.1133	0.0894	0.0%	1.12 [0.94, 1.33]
Kaleta 2019	-0.0101	0.0961	11.2%	0.99 [0.82, 1.20]
Mohammed 2020	-0.1744	0.8151	2.6%	0.84 [0.17, 4.15]
Rodriguez 2017	0.0488	0.2069	9.6%	1.05 [0.70, 1.58]
Sutfin 2013	0.6043	0.1986	9.7%	1.83 [1.24, 2.70]
Thrasher 2016	1.4609	0.1004	11.2%	4.31 [3.54, 5.25]
**Total (95% CI)**			**100.0%**	**2.16 [1.62, 2.90]**

Heterogeneity: Tau² = 0.19; Chi² = 188.03, df = 9 (P < 0.00001); I² = 95%
Test for overall effect: Z = 5.18 (P < 0.00001)

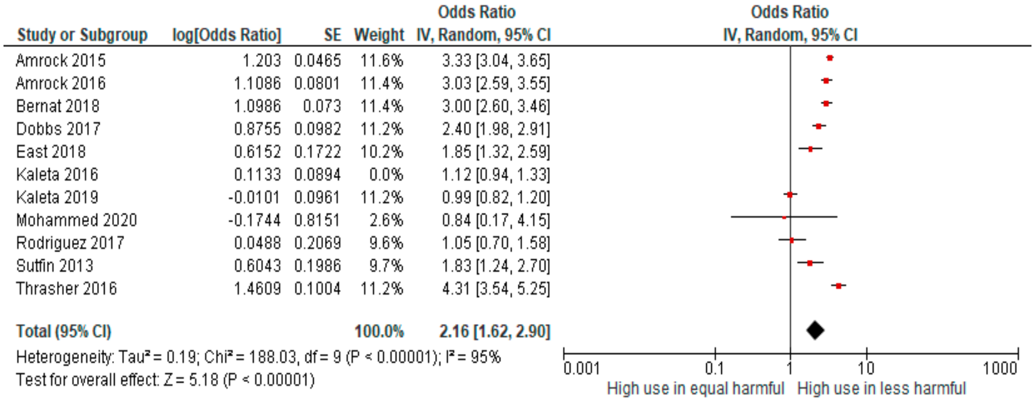

(c) Perceived harmfulness (more harmful vs equal harmful)

Study or Subgroup	log[Odds Ratio]	SE	Weight	Odds Ratio IV, Random, 95% CI
Amrock 2015	0.2776	0.093	14.2%	1.32 [1.10, 1.58]
Amrock 2016	0.392	0.1608	12.7%	1.48 [1.08, 2.03]
Bernat 2018	-0.2877	0.1582	12.8%	0.75 [0.55, 1.02]
Dobbs 2017	-0.3147	0.2356	10.8%	0.73 [0.46, 1.16]
East 2018	0.1655	0.4589	6.0%	1.18 [0.48, 2.90]
Kaleta 2016	1.203	0.1362	0.0%	3.33 [2.55, 4.35]
Kaleta 2019	0.1484	0.1647	12.6%	1.16 [0.84, 1.60]
Mohammed 2020	-1.1712	0.8379	2.5%	0.31 [0.06, 1.60]
Rodriguez 2017	0.7701	0.3261	8.5%	2.16 [1.14, 4.09]
Sutfin 2013	0.2231	0.4282	6.5%	1.25 [0.54, 2.89]
Thrasher 2016	-0.5276	0.127	13.5%	0.59 [0.46, 0.76]
**Total (95% CI)**			**100.0%**	**1.03 [0.77, 1.36]**

Heterogeneity: Tau² = 0.14; Chi² = 45.62, df = 9 (P < 0.00001); I² = 80%
Test for overall effect: Z = 0.19 (P = 0.85)

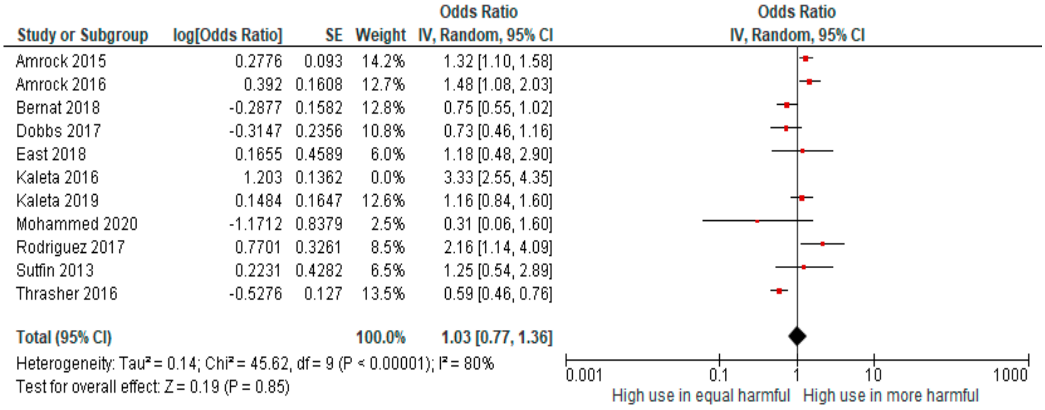

(d) Perceived addictiveness (disagree vs agree)

Study or Subgroup	log[Odds Ratio]	SE	Weight	Odds Ratio IV, Random, 95% CI
Aly 2018	1.7246	0.2235	21.7%	5.61 [3.62, 8.69]
Andrews 2016	0.3507	0.1211	25.7%	1.42 [1.12, 1.80]
Bernat 2018	-3.5066	0.2069	0.0%	0.03 [0.02, 0.05]
Cooper 2016	0.3988	0.119	25.8%	1.49 [1.18, 1.88]
Cooper 2018	0.2311	0.0786	26.9%	1.26 [1.08, 1.47]
**Total (95% CI)**			**100.0%**	**1.88 [1.21, 2.90]**

Heterogeneity: Tau² = 0.18; Chi² = 39.85, df = 3 (P < 0.00001); I² = 92%
Test for overall effect: Z = 2.83 (P = 0.005)

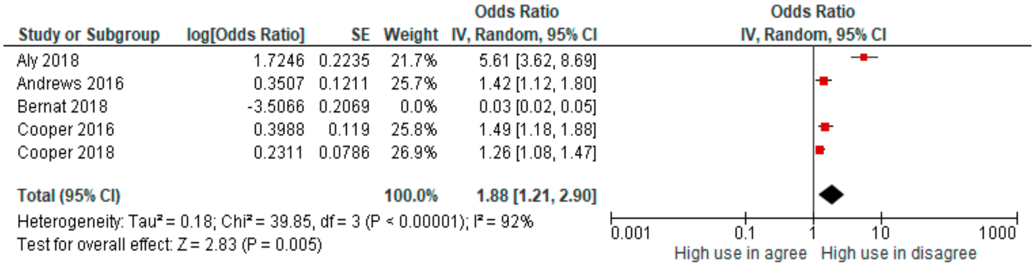

(e) Perceived addictiveness (less addictive vs equal addictive)

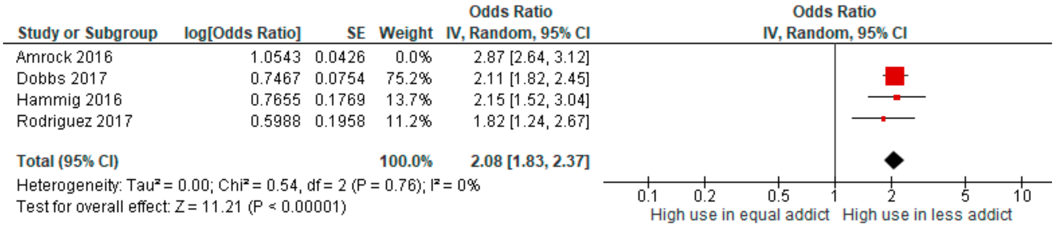

(f) Perceived addictiveness (more addictive vs equal addictive)

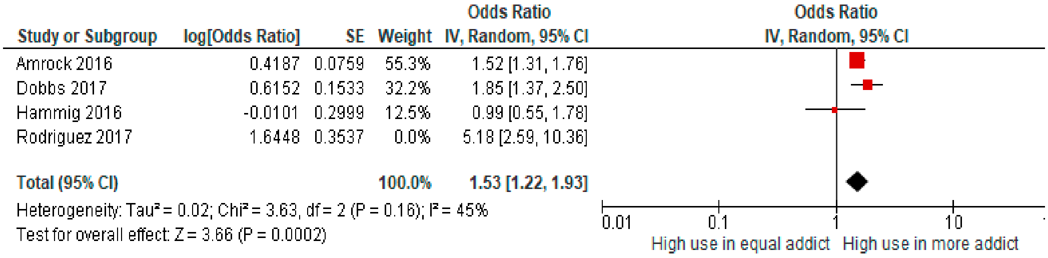

Figure S1 Forest plot of the meta-analysis for perceived risks, (a-c) perceived harmfulness, (d-f) perceived addictiveness and ever e-cigarettes use in young people after sensitivity analysis. Each study is identified by their first author. The individual effect estimates is identified as odds ratios with lower and upper limits (95% confidence interval)

Supplementary Table S4 Quality assessment of the present review (GRADE Evidence Profile)

Certainty assessment							№ of patients		Effect		Certainty	Importance
**No of studies**	**Study design**	**Risk of bias**	**Inconsistency**	**Indirectness**	**Imprecision**	**Other considerations**	**Disagree to perceived harmfulness**	**Agree to perceived harmfulness**	**Relative (95% CI)**	**Absolute (95% CI)**		
**Question**: Should **disagree vs. agree** to **perceived harmfulness** be used for preventing e-cigarette use in young people?												
7	observational studies	not serious	serious [a]	not serious	not serious	Strong association			**OR 2.20** (1.41 to 3.43)	**2 fewer per 1,000** (from 3 fewer to 1 fewer)	⊕⊕◯◯ LOW	
**Certainty assessment**							**№ of patients**		**Effect**		**Certainty**	**Importance**
**No of studies**	**Study design**	**Risk of bias**	**Inconsistency**	**Indirectness**	**Imprecision**	**Other considerations**	**Perceived less harmful**	**Perceived equally harmful**	**Relative (95% CI)**	**Absolute (95% CI)**		
**Question**: Should **less harmful vs. equally harmful** to tobacco cigarettes be used for preventing e-cigarette use in young people?												
11	observational studies	not serious	serious [a]	not serious	not serious	strong association			**OR 2.01** (1.47 to 2.75)	**2 fewer per 1,000** (from 3 fewer to 1 fewer)	⊕⊕◯◯ LOW	
**Certainty assessment**							**№ of patients**		**Effect**		**Certainty**	**Importance**
**No of studies**	**Study design**	**Risk of bias**	**Inconsistency**	**Indirectness**	**Imprecision**	**Other considerations**	**Perceived more harmful**	**Perceived equally harmful**	**Relative (95% CI)**	**Absolute (95% CI)**		
**Question**: Should **more harmful vs. equally harmful** to tobacco cigarettes be used for preventing e-cigarette use in young people?												
11	observational studies	not serious	serious [a]	not serious	not serious	none			**OR 1.16** (0.80 to 1.68)	**1 fewer per 1,000** (from 2 fewer to 1 fewer)	⊕◯◯◯ VERY LOW	

Certainty assessment							№ of patients		Effect		Certainty	Importance
**No of studies**	**Study design**	**Risk of bias**	**Inconsistency**	**Indirectness**	**Imprecision**	**Other considerations**	**Disagree to perceived addictiveness**	**Agree to perceived addictiveness**	**Relative (95% CI)**	**Absolute (95% CI)**		
**Question**: Should **disagree vs. agree** to **perceived addictiveness** be used for preventing e-cigarette use in young people?												
5	observational studies	not serious	serious [a]	not serious	not serious	none			**OR 0.86** (0.29 to 2.56)	**1 fewer per 1,000** (from 3 fewer to 0 fewer)	⊕◯◯◯ VERY LOW	

Certainty assessment							№ of patients		Effect		Certainty	Importance
**No of studies**	**Study design**	**Risk of bias**	**Inconsistency**	**Indirectness**	**Imprecision**	**Other considerations**	**Perceived less addictive**	**Perceived equally addictive**	**Relative (95% CI)**	**Absolute (95% CI)**		
**Question**: Should **less addictive vs. equally addictive** to tobacco cigarettes be used for preventing e-cigarette use in young people?												
4	observational studies	not serious	serious [a]	not serious	not serious	strong association	0/0	0/0	**OR 2.28** (1.81 to 2.88)	**2 fewer per 1,000** (from 3 fewer to 2 fewer)	⊕⊕◯◯ LOW	

Certainty assessment							№ of patients		Effect		Certainty	Importance
**No of studies**	**Study design**	**Risk of bias**	**Inconsistency**	**Indirectness**	**Imprecision**	**Other considerations**	**Perceived more addictive**	**Perceived equally addictive**	**Relative (95% CI)**	**Absolute (95% CI)**		
**Question**: Should **more addictive vs. equally addictive** to tobacco cigarettes be used for preventing e-cigarette use in young people?												
4	observational studies	not serious	serious [a]	not serious	not serious	none	0/0	0/0	**OR 1.82** (1.22 to 2.73)	**2 fewer per 1,000** (from 3 fewer to 1 fewer)	⊕◯◯◯ VERY LOW	

Question: Perceived harmfulness compared to traditional cigarettes (**perceived risk**) for preventing ever e-cigarette use across **age group**

Certainty assessment							№ of patients		Effect		Certainty	Importance
**No of studies**	**Study design**	**Risk of bias**	**Inconsistency**	**Indirectness**	**Imprecision**	**Other considerations**	**Perceived harmfulness**	**[comparison]**	**Relative (95% CI)**	**Absolute (95% CI)**		
Perceived less harmful and ever e-cigarette use (Adolescents)												
9	observational studies	not serious	serious [a]	not serious	not serious	strong association			**OR 2.18** (1.55 to 3.07)	**2 fewer per 1,000** (from 3 fewer to 2 fewer)	⊕⊕◯◯ LOW	
Perceived less harmful and ever e-cigarette use (Young Adults)												
2	observational studies	not serious	not serious	not serious	not serious	none			**OR 1.39** (0.81 to 2.40)	**1 fewer per 1,000** (from 2 fewer to 1 fewer)	⊕⊕◯◯ LOW	
Perceived more harmful and ever e-cigarette use (Adolescents)												
9	observational studies	not serious	serious [a]	not serious	not serious	none			**OR 1.08** (0.72 to 1.63)	**1 fewer per 1,000** (from 2 fewer to 1 fewer)	⊕◯◯◯ VERY LOW	
Perceived more harmful and ever e-cigarette use (Young Adults)												
2	observational studies	not serious	not serious	not serious	not serious	none			**OR 1.76** (1.05 to 2.96)	**2 fewer per 1,000** (from 3 fewer to 1 fewer)	⊕⊕◯◯ LOW	

GRADE Working Group grades of evidence
High quality: Further research is very unlikely to change our confidence in the estimate of effect
Moderate quality: Further research is likely to have an important impact on our confidence in the estimate of effect and may change the estimate
Low quality: Further research is very likely to have an important impact on our confidence in the estimate of effect and is likely to change the estimate
Very low quality: We are very uncertain about the estimate

CI: Confidence interval; **OR:** Odds ratio

a. The quality of evidence was downgraded due to considerable heterogeneity; majority of studies are cross-sectional studies
